# Clinical Characteristics of 6578 Adult Patients With Cholera Admitted to Community and Referral Cholera Treatment Centers in Lusaka, Zambia, October 2023 to April 2024

**DOI:** 10.1093/ofid/ofaf277

**Published:** 2025-05-08

**Authors:** Nyuma Mbewe, Tadatsugu Imamura, Suilanji Sivile, Annel Sinkala, Paul Zulu, Chitalu Chanda, Neil Naik, Nawa Kalima, Roy Tepa, Kabaso Mwewa, Kenneth Kapololwe, Anchindika Mugala, Aggrey Mweemba, Davie Simwaba, Muzala Kapina, Kelvin Mwangilwa, Lalisa Nambeya, Sophia Msiska, Aspha Choonga, Bob Chirwa, Shingo Mitsushima, Yuuki Tsuchihashi, Nathan Kapata, Taro Kamigaki, Lloyd Mulenga, Roma Chilengi

**Affiliations:** Cholera Incident Management System, Zambia National Public Health Institute, Lusaka, Zambia; Project for Strengthening Laboratory-based Surveillance for Infectious Diseases, Japan International Cooperation Agency, Tokyo, Japan; Center for Postgraduate Education and Training, National Center for Child Health and Development, Tokyo, Japan; Adult Infectious Disease Center, University Teaching Hospital, Lusaka, Zambia; Adult Infectious Disease Center, University Teaching Hospital, Lusaka, Zambia; Cholera Incident Management System, Zambia National Public Health Institute, Lusaka, Zambia; Adult Infectious Disease Center, University Teaching Hospital, Lusaka, Zambia; Adult Infectious Disease Center, University Teaching Hospital, Lusaka, Zambia; National Heart Hospital, Lusaka, Zambia; Department of Infectious Diseases, Levy Mwanawasa University Teaching Hospital, Lusaka, Zambia; Department of Infectious Diseases, Levy Mwanawasa University Teaching Hospital, Lusaka, Zambia; Department of Infectious Diseases, Levy Mwanawasa University Teaching Hospital, Lusaka, Zambia; Department of Infectious Diseases, Levy Mwanawasa University Teaching Hospital, Lusaka, Zambia; Department of Infectious Diseases, Levy Mwanawasa University Teaching Hospital, Lusaka, Zambia; Cholera Incident Management System, Zambia National Public Health Institute, Lusaka, Zambia; Cholera Incident Management System, Zambia National Public Health Institute, Lusaka, Zambia; Cholera Incident Management System, Zambia National Public Health Institute, Lusaka, Zambia; Lusaka District Health Office, Lusaka, Zambia; Luska Provincial Health Office, Lusaka; Luska Provincial Health Office, Lusaka; Adult Infectious Disease Center, University Teaching Hospital, Lusaka, Zambia; Center for Field Epidemic Intelligence, Research and Professional Development, National Institute of Infectious Diseases, Tokyo, Japan; Center for Field Epidemic Intelligence, Research and Professional Development, National Institute of Infectious Diseases, Tokyo, Japan; Cholera Incident Management System, Zambia National Public Health Institute, Lusaka, Zambia; Center for Surveillance, Immunization, and Epidemiologic Research, National Institute of Infectious Diseases, Tokyo, Japan; Adult Infectious Disease Center, University Teaching Hospital, Lusaka, Zambia; Cholera Incident Management System, Zambia National Public Health Institute, Lusaka, Zambia

**Keywords:** adult patients, case fatality rate, cholera treatment centers, outbreak response, referral system, vibrio cholerae

## Abstract

**Background:**

Zambia experienced the largest cholera outbreak in the country history in 2023–2024 in the capital, Lusaka. This study aimed to identify the clinical characteristics of the adult patients hospitalized at the community and referral cholera treatment centers (CTCs) to determine factors associated with their severe clinical outcomes during the outbreak.

**Methods:**

Clinical information on the adult patients with cholera in the community and referral CTCs was retrospectively analyzed. Clinical factors associated with the fatal outcome were explored by multivariate analysis, using Firth's penalized logistic regression.

**Results:**

A total of 6578 adult cases were identified. The overall case fatality rate was 1.0% (51 of 5020), and it was highest in a referral CTC specializing in patients with underlying conditions (4.1% [32 of 772]). In the multivariate analysis, age (odds ratio, 1.05 [95% confidence interval, 1.02–1.09]), human immunodeficiency virus infections (5.68 [2.12–15.30]), diabetes mellitus (8.21 [1.38–34.00]), and severe dehydration at admission (5.97 [1.29–56.94]) were independently correlated with fatal outcomes.

**Conclusions:**

Clinical factors, including age, underlying conditions, and disease severity at admission, were shown to be associated with severe clinical outcomes in adult patients with cholera. Enhanced case management strategies targeted for such high-risk groups might be beneficial in reducing the case fatality rate during cholera outbreaks.

## Teaser

Age, underlying conditions, and disease severity at admission were shown to be associated with fatal outcomes of adult patients during the cholera outbreak in Lusaka, Zambia, 2023–24, which highlights the importance of targeted intervention for such high-risk groups.

Cholera is an acute diarrheal disease caused by *Vibrio cholerae* infection [1]. It affects both adults and children, and severe symptoms (eg, hypovolemic shock and electrolyte loss) develop in approximately 5%–10% of patients [2]. Fatality is known to be more prevalent among individuals with risk factors (eg, age, comorbid conditions, and poor nutritional status) [2]. In well-controlled cholera outbreaks, the case fatality rate (CFR)—the number of fatal cases among of the total number of cholera cases—is expected to be <1% [2]. However, the CFRs vary between outbreaks in different times and regions, depending on multiple factors, including the sociomedical and metrological characteristics of the residents in the affected areas, public health preparedness system and case management capacity, and the intensity of the cholera transmission [3, 4]. Today, it is estimated that 1.3–4.0 million cases and 21 000–143 000 deaths of cholera are reported annually worldwide [5].

Zambia is a cholera-endemic country that has experienced >30 outbreaks between 1977 and 2019 [6]. Notably, the most recent cholera outbreak, which started from Lusaka District in October 2023, included the largest recorded number of patients in the country's history [6, 7]. ln Lusaka, the first cholera case was reported in one of the high-density periurban area of the city (ie, Kanyama subdistrict) on 15 October 2023; this had been the incident case of the recent cholera outbreak in the area [8, 9]. The number of cases increased rapidly across Lusaka Province toward the peak of the epidemics in early January 2024 [10].

During the outbreak, the national cholera incident management system (IMS), established by the Ministry of Health, Zambia (MOH) and the Zambia National Public Health Institute (ZNPHI), set up multiple cholera treatment centers (CTCs) in the community and referral healthcare facilities in the outbreak area to manage patients who required clinical care and hospitalization. Adult patients were assessed for disease severity and risk factors for severe diseases (eg, underlying conditions) at any CTCs where they were first admitted to, and severe cases and those with risks were strategically centralized and managed in the referral CTCs. This strategy prevented the primary and secondary healthcare resources from being overwhelmed by the surge of cholera cases during the outbreak. To date, the clinical characteristics of these adult patients in the CTCs have not been formally documented, and it remains unknown which patient characteristics may predispose infected persons to severe outcomes in the adult population. Therefore, the current study aimed to determine the clinical characteristics of the adult patients hospitalized at the community and referral CTCs and to report factors associated with severe clinical outcomes during the outbreak in Lusaka, Zambia, between October 2023 and April 2024.

## METHODS

### Study Design

We analyzed data from patients with cholera, which were collected from the community and referral cholera treatment centers (CTC) in Lusaka, Zambia, between 15 October 2023 and 30 April 2024. Zambia is a land-locked country, and its capital Lusaka District has a population of >2 200 000. The rainy season starts in November and ends in April. The precipitation averages 650 mm per year, but the heaviest rain falls during January, when the rainfall averages 220 mm, causing severe flooding in most parts due to lack of or clogged drainage. With the floods come epidemics of enteric pathogens, especially in the low-income residential areas in Lusaka.

### CTC Details

During the 2023–2024 cholera outbreak in Lusaka, a total of 8 CTCs were operationalized in Lusaka, by the cholera IMS organized by the MOH and ZNPHI. Six CTCs opened in the public first-level hospitals and community health centers (ie, Kanayma, Chawama, Chipata, and Matero Level One Hospitals, Bauleni Mini Hospital, and George Health Center) and served as the community CTCs, which provided hospital visits and admission of patients from their catchment areas. In addition to the community CTCs, 2 specialized CTCs were opened, one in a public third-level (tertiary) hospital, the Levy Mwanawasa University Teaching Hospital (LMUTH), and another in the National Heroes Stadium. The CTC in the LMUTH (LMUTH CTC) was opened on 18 December 2023 and served patients with cholera and underlying conditions. The CTC in the National Heroes Stadium (Heroes CTC) served as a high-volume CTC, with a capacity of >1000 patient beds [11].

Patients first visited or were admitted to the community CTCs, where they were clinically assessed for current disease severity and risk of severe outcomes. Patients who had underlying conditions or presented with severe symptoms were referred to the LMUTH CTC after stabilization. After the opening of the Heroes CTC, patients with cholera, particularly those presenting with severe symptoms, were referred there when the community CTCs were overwhelmed with the surge of cases in their catchment area.

### Clinical Management

**Figure 1.**
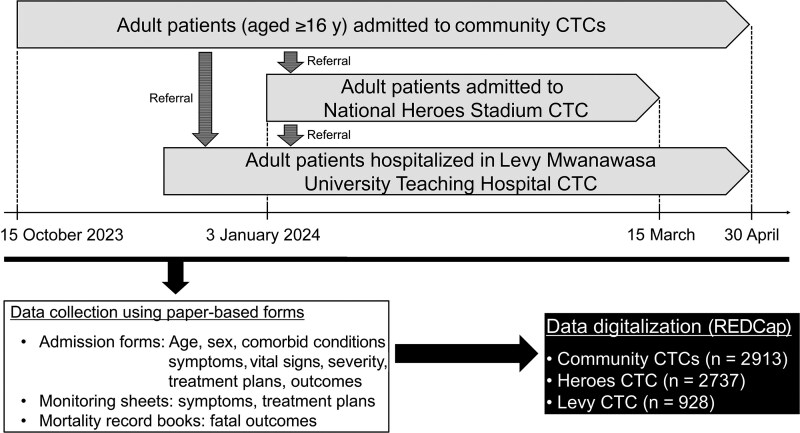
Collection and digitalization of clinical information of adult patients with suspected cholera admitted to community and referral cholera treatment centers (CTCs) in Lusaka, Zambia, between October 2023 and April 2024.

Suspected cholera cases were defined as those in individuals who presented with ≥3 bouts of watery stools within 24 hours [12]. Among the suspected case patients, those whose stool samples tested positive for *Vibrio cholerae* by bacterial culture or polymerase chain reaction were categorized as having confirmed cases. During the outbreak, CTCs in Lusaka accepted patients with both suspected and confirmed cholera cases. At admission, all patients were evaluated for clinical presentation and severity to determine the treatment, based on plans A–C; A (oral rehydration solution; ORS over 4 hours), B (ORS over 4 hours, 75ml/kg), and C (intravenous Rinder’s lactate), respectively [12]. Patients with no dehydration were monitored every 6 hours, and those with some or severe dehydration were monitored every 2 hours. Patient characteristics and treatment plans were recorded in the paper-based admission forms, which were generated by the case management pillar of the national cholera IMS in reference to the Global Task Force on Cholera Control treatment guidelines (Figure 1) [13].

### Clinical Data Collection

Patient clinical presentations and new treatment plans based on them were first recorded in the paper-based monitoring sheets (Figure 1). Patient clinical outcomes were recorded either on the admission forms or in the mortality record books (Figure 1). After discharge or death of the patients, these paper forms and books were stored in the data management room in each CTC. Patient information recorded on these paper forms and books was retrospectively reviewed and digitalized by trained data collectors, using REDCap software. Encoded data were exported as Microsoft Excel sheets and used as the master dataset for analysis. Patients whose paper forms and book records could not be accessed by the data encoders were excluded from this study.

### Data Analysis

Descriptive analysis was conducted for patient characteristics and recorded in the master dataset. Continuous variables were summarized as median values with interquartile range. Categorical values were summarized as proportions of patients with respective characteristics. Records of treatment plans during hospitalization were summarized in a flow chart. Patients were regarded as having experienced an escalation in treatment plan when treatment plan was changed from A to B, A to C, or B to C during hospitalization.

The temporal distribution of the patients with cholera included in the study was summarized in an epidemiological curve based on the date of admission. CFRs were calculated as the number of fatal cases divided by the total number of cases in the group and were calculated in total and for specific patient groups of interests.

### Human Resource Data

The number of healthcare workers in each CTC was collected by reviewing the record of the staff in shifts at Kanayma Level One Hospital (15 October 2023 to 31 March 2024), Chawama Level One Hospital (1 November 2023 to 19 April 2024), Chipata Level One Hospital (22 October 2023 to 31 March 2024), Matero Level One Hospital (13 January to 31 March 2024), Bauleni Mini Hospital (15 October 2023 to 31 March 2024), and George Health Center (15 October 2023 to 31 March 2024). The temporal distribution of the healthcare workers was shown in bar graphs by type of healthcare professionals and by CTC based on the date when the staff was on shift.

### Statistical Analysis

Statistical analysis was conducted using R software, version 3.5.0 (R Foundation for Statistical Computing), with χ^2^ and Fisher exact tests used to compare categorical variables between patients of 2 groups and Student *t* and Wilcoxon rank sum tests used to compare the patient characteristics as continuous variables.

In the multivariate analysis, associations between the fatal outcome as the dependent variable and patient characteristics, including age, sex, underlying conditions, and disease severity at admission, as the independent variables were evaluated, using Firth's penalized logistic regression. Odds ratios (ORs) were calculated for crude and multivariate analyses and were presented 95% confidence intervals (CIs). Differences were considered statistically significant at *P* < .05.

### Ethical Considerations

All data analyzed in this study were anonymized, with no personally identifiable data that can be linked to an individual patient.

Institutional consent to use patient records was obtained from the MOH. The secondary use of patient data (collected as part of the emergency medical response of MOH and ZNPHI) for analysis and publication was approved by the National Health Research Authority (reference NHRA-1803/18/12/2024).

## RESULTS

### Patient Characteristics

Patient information was available for a total of 6578 patients with cholera, including 2913 in the community and 3665 in the referral CTCs (Figure 1 and Supplementary Table 1). The most common age group was 20–29 years, followed by 30–39 years in both community and referral CTCs (Table 1). Male patients were overrepresented compared with female patients (Table 1). The most common underlying conditions included human immunodeficiency virus (HIV) infections (Table 1). The proportion of patients with severe dehydration at admission was highest in the community CTCs (27.3% [781 of 2863]), followed by the LMUTH CTC (23.8% [218 of 910]) (Table 1). More than 90% of patients with severe dehydration were administered treatment plan C at admission in both the community and referral CTCs (Supplementary Table 2). The total CFR was 1.0% (51 of 5020), which was higher in the referral than the community CTCs, 4.1% (32 of 772) in the LMUTH CTC and 1.3% (34 of 2574) in the Heroes CTC (Table 1 and Supplementary Table 3). More than 99% of the patients in the community and the Heroes CTCs recovered and were discharged, as did >95% in the LMUTH CTC (Table 1).

**Table 1.** Characteristics of 6578 Adult Patients Hospitalized at Cholera Treatment Centers in Lusaka, Zambia, 15 October 2023 to 30 April 2024

Characteristic	Patients at Community CTCs, No. (%) (n = 2913)	Patients at Referral CTCs, No. (%)		
		Total (n = 3665)	Heroes Stadium (n = 2737)	LMUTH (n = 928)
Age group, y				
16–19	256 (8.8)	257 (7.0)	201 (7.3)	56 (6.0)
20–29	1218 (41.8)	1371 (37.4)	1052 (38.4)	319 (34.4)
30–39	764 (26.2)	1009 (27.5)	742 (27.1)	267 (28.8)
40–49	390 (13.4)	590 (16.1)	442 (16.1)	148 (15.9)
50–59	176 (6.0)	245 (6.7)	180 (6.6)	65 (7.0)
60–69	61 (2.1)	117 (3.2)	79 (2.9)	38 (4.1)
70–79	35 (1.2)	55 (1.5)	34 (1.2)	21 (2.3)
80–89	9 (0.3)	18 (0.5)	7 (0.3)	11 (1.2)
90–99	4 (0.1)	3 (0.1)	0 (0)	3 (0.3)
Sex				
Female	1149 (39.6)	1558 (42.6)	1114 (40.8)	444 (47.9)
Male	1756 (60.4)	2101 (57.4)	1618 (59.2)	483 (52.1)
Underlying medical conditions				
HIV infection	159 (9.6)	278 (19.7)	127 (13.5)	151 (32.1)
Hypertension	97 (3.3)	97 (2.6)	19 (0.7)	78 (8.4)
DM	19 (0.7)	43 (1.2)	7 (0.3)	36 (3.9)
Tuberculosis	8 (0.3)	18 (0.5)	0 (0)	18 (1.9)
Epilepsy/seizures	2 (0.1)	10 (0.3)	0 (0)	10 (1.1)
Liver disease	1 (0.0)	8 (0.2)	0 (0)	8 (0.9)
Kidney disease	0 (0)	7 (0.2)	0 (0)	7 (0.8)
Alcohol dependency	1 (0.0)	6 (0.2)	0 (0)	6 (0.6)
Anemia	2 (0.1)	3 (0.1)	0 (0)	3 (0.3)
History of oral cholera vaccine before admission	177 (9.9)	126 (8.3)	122 (10.9)	4 (1.0)
Disease severity at admission				
No dehydration	996 (34.8)	992 (32.5)	734 (34.2)	258 (28.4)
Some dehydration	1086 (37.9)	1560 (51.1)	1123 (52.4)	437 (48.0)
Severe dehydration	781 (27.3)	502 (16.4)	287 (13.4)	215 (23.6)
Initial treatment plan at admission				
A	989 (34.2)	942 (31.5)	693 (33.3)	249 (27.2)
B	1104 (38.2)	1548 (51.7)	1100 (52.9)	448 (49.0)
C	796 (27.6)	503 (16.8)	285 (13.7)	218 (23.8)
Outcome				
Discharge	2429 (99.3)	2540 (98.7)	1800 (99.9)	740 (95.9)
Death	17 (0.7)	34 (1.3)	2 (0.1)	32 (4.1)

Abbreviations: CTCs, community treatment centers; DM, diabetes mellitus; HIV, human immunodeficiency virus; LMUTH, Levy Mwanawasa University Teaching Hospital. Treatment plans A; oral rehydration solution; ORS over 4 hours, B (ORS over 4 hours, 75ml/kg), and C (intravenous Rinder's lactate)” [12].

**Figure 2.**
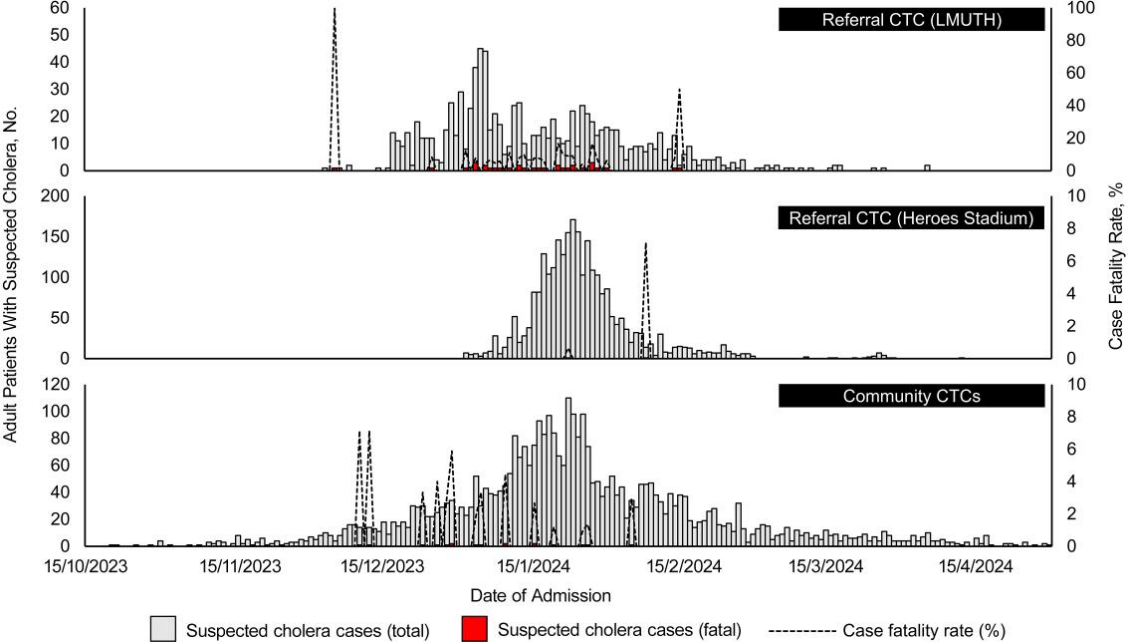
Temporal distribution of adult patients hospitalized at cholera treatment centers (CTCs) in Lusaka, Zambia, between 15 October 2023 and 30 April 2024. Abbreviation: LMUTH, Levy Mwanawasa University Teaching Hospital.

The number of patients in the community CTCs increased rapidly after mid-November and peaked on 22 January 2024 (Figure 2). The number of patients hospitalized at the Heroes Stadium showed a similar pattern (Figure 2). New admissions at the community and the Heroes CTCs decreased gradually toward late February (Figure 2). The LMUTH CTC continuously accepted patients with <30 samples per day, except between 3 and 5 January 2024, when it recorded its peak (Figure 2).

### Clinical Course During Hospitalization

**Figure 3.**
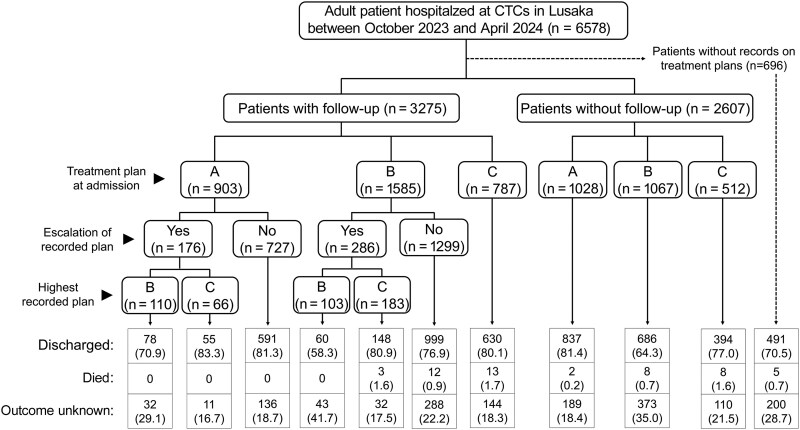
Patient treatment plans and outcomes in adult patients hospitalized at cholera treatment centers (CTCs) in Lusaka, Zambia, between 15 October 2023 and 30 April 2024. Treatment plans A; oral rehydration solution; ORS over 4 hours, B (ORS over 4 hours, 75ml/kg), and C (intravenous Rinder’s lactate)" [12].

Among the 6578 adult patients, records of the treatment plan at admission and follow-up details during hospitalization were available for 3275 (50.0%) (Figure 3). Among 1585 patients on treatment plan B at admission, 286 (18.0%) experienced an escalation of plan, and 3 of the patients escalated to plan C later died (1.6% [3 of 183]). Although 1299 patients on treatment plan B at admission had no record of an escalation in plan, 12 (0.9%) of them died (Figure 3). Among 787 patients on treatment plan C at admission, 13 (1.7%) died during hospitalization (Figure 3).

Among 2607 patients without records of follow-up details during hospitalization, deaths were reported among those on treatment plans A (0.2% [2 of 1028]), B (0.7% [8 of 1067]), and C (1.6% [8 of 512]). Of 696 patients without any record of the treatment plans at admission or follow-up details, deaths occurred in 5 (0.7%).

### Characteristics of Patients With Fatal Outcomes

Outcomes were recorded for 5020 of the 6578 adult patients (76.3%) (Table 2). The median age (interquartile range) of the 51 patients who died was 38.0 (30.0–49.5) years, significantly older than in the 4696 who were discharged (30.0 [24.0–39.0] years; *P* < .001) (Table 2). The proportion of male patients was significantly higher among those who died (78.4% [40 of 51]) than among those who were discharged (57.6% [2856 of 4960; *P* = .003). Among the underlying conditions, HIV infection, hypertension, diabetes mellitus (DM), liver diseases, and alcohol dependency were more prevalent among the patients who died.

**Table 2.** Characteristics by Outcome Among Adult Patients at Referral Cholera Treatment Centers in Lusaka, Zambia, 15 October 2023 to 30 April 2024

Characteristic	Patients by Outcome, No. (%)^a^				*P* Value Comparing Outcomes^b^
	Known Outcome (n = 5020)	Unknown Outcome (n = 1558)	Death (n = 51)	Discharge (n = 4969)	
Age, median (IQR), y	30.0 (24.0–40.0)	31.0 (24.0–42.0)	38.0 (30.0–49.5)	30.0 (24.0–39.0)	<.001
Sex					
Female	2115 (42.2)	592 (38.1)	11 (21.6)	2104 (42.4)	.003
Male	2896 (57.8)	961 (61.9)	40 (78.4)	2856 (57.6)	
Underlying medical conditions					
HIV infection	324 (12.6)	113 (37.9)	8 (47.1)	316 (12.3)	<.001
Hypertension	157 (3.1)	37 (2.4)	6 (11.8)	151 (3.0)	.005
DM	47 (.9)	15 (1.0)	4 (7.8)	43 (0.9)	.001
Tuberculosis	23 (0.5)	3 (0.2)	1 (2.0)	22 (0.4)	.21
Epilepsy/seizures	8 (0.2)	4 (0.3)	0 (0)	8 (0.2)	>.99
Liver disease	8 (0.2)	1 (0.1)	2 (4.0)	6 (0.1)	.003
Kidney disease	6 (0.1)	1 (0.1)	1 (2.0)	5 (0.1)	.06
Alcohol dependency	4 (0.1)	3 (0.2)	1 (2.0)	3 (0.1)	.04
Anemia	3 (0.1)	2 (0.1)	0 (0)	3 (0.1)	>.99
History of oral cholera vaccine before admission	222 (8.1)	81 (14.1)	1 (6.3)	221 (8.2)	>.99
Duration of diarrhea in present illness, median (IQR), d	1.0 (1.0–2.0)	2.0 (1.0–2.0)	1.5 (1.0–3.0)	1.0 (1.0–2.0)	.09
CTC type					
Community	2446 (48.7)	467 (30.0)	17 (33.3)	2429 (48.9)	.03
Referral	2574 (51.3)	1091 (70.0)	34 (66.7)	2540 (51.1)	
Symptoms at admission					
Sunken eyes	2766 (55.1)	845(54.2)	34 (66.7)	3732 (75.1)	.12
Skin goes back slowly	1485 (29.6)	472 (30.3)	11 (21.6)	1474 (29.7)	.28
Lethargic	855 (17.0)	229 (14.7)	19 (37.3)	836 (16.8)	>.99
Drinks eagerly	911 (18.1)	263 (16.9)	4 (7.8)	907 (18.3)	.07
Irritable	407 (8.1)	85 (5.5)	7 (13.7)	400 (8.0)	.19
Not drinking	448 (8.9)	85 (5.5)	16 (31.4)	432 (8.7)	<.001
Unconscious	36 (0.7)	12 (0.8)	2 (3.9)	34 (0.7)	.051
Vital signs at admission, median (IQR)					
Temperature, °C	36.3 (36.0–36.7)	36.3 (36.0–36.7)	36.2 (36.0–36.7)	36.3 (36.0–36.7)	<.001
Pulse rate per min	88.0 (78.0–100.0)	88.0 (77.0–99.0)	89.0 (75.0–107.0)	88.0 (78.0–100.0)	<.001
Respirations/min	20.0 (18.0–22.0)	20.0 (18.0–22.0)	20.0 (18.0–22.0)	20.0 (18.0–22.0)	.02
Systolic BP, mm Hg	116.0 (105.0–126.5)	117.0 (105.0–127.0)	110.5 (101.2–120.0)	116.0 (105.0–127.0)	<.001
Diastolic BP, mm Hg	77.0 (68.0–85.0)	77.0 (68.0–86.5)	71.5 (61.3–80.0)	77.0 (68.0–85.0)	<.001
Spo_2_	98.0 (96.0–99.0)	98.0 (96.0–99.0)	98.0 (95.3–98.0)	98.0 (96.0–99.0)	.01
Disease severity at admission					
No dehydration	1607 (35.0)	381 (28.8)	4 (8.7)	1603 (35.3)	<.001
Some dehydration	1955 (42.6)	691 (52.2)	22 (47.8)	1933 (42.5)	
Severe dehydration	1031 (22.4)	252 (19.0)	20 (43.5)	1011 (22.2)	
Initial treatment plan at admission					
A	1563 (34.5)	368 (27.1)	2 (4.3)	1561 (34.9)	<.001
B	1916 (42.4)	736 (54.2)	23 (50.0)	1893 (42.3)	
C	1045 (23.1)	254 (18.7)	21 (45.7)	1024 (22.9)	
Escalation of treatment plan during hospitalization	561 (18.6)	156 (19.7)	4 (14.3)	557 (18.7)	.81

Abbreviations: BP, blood pressure; CTC, cholera treatment center; DM, diabetes mellitus; HIV, human immunodeficiency virus; IQR, interquartile range; Spo_2_, peripheral oxygen saturation. Treatment plans A; oral rehydration solution; ORS over 4 hours, B (ORS over 4 hours, 75ml/kg), and C (intravenous Rinder's lactate)” [12].

^a^Data represent no. (%) of patients unless otherwise specified.

^b^ *P* values for comparisons between the 2 outcome groups (death and discharge), with χ^2^ and Fisher exact tests used to compare categorical variables and Student *t* or Wilcoxon rank sum tests used to compare continuous variables.

### Risk Factors for Fatal Outcome

In the bivariate analysis, variables positively correlated with a fatal outcome included age (OR, 1.04 [95% CI, 1.02–1.06), male sex (2.68 [1.42–5.50]), HIV-positive status (6.32 [2.36–16.66]), hypertension (4.25 [1.61–9.39]), DM (9.75 [2.85–25.33]), some dehydration at admission (9.48 [2.80–59.16]), and severe dehydration at admission (16.01 [4.68–100.22]) (Table 3). In the multivariate analysis, age (OR, 1.05 [95% CI, 1.02–1.09]), HIV-positive status (5.68 [CI 2.12–15.30]), DM (8.21 [1.38–34.00]), and severe dehydration at admission (5.97 [1.29–56.94]) were independently correlated with a fatal outcome.

**Table 3.** Characteristics Associated With Fatal Outcomes Among Adult Patients Hospitalized at Referral Cholera Treatment Centers in Lusaka, Zambia, 15 October 2023 to 30 April 2024

Characteristic	Category	Association With Fatal Outcome^a^			
		Crude Analysis		PLR Analysis	
		OR (95% CI)	*P* Value	OR (95% CI)	*P* Value
Age (y)		1.04 (1.02–1.06)	<.001	1.05 (1.02–1.09)	.004
Sex	Female	Ref	Ref	Ref	Ref
	Male	2.68 (1.42–5.50)	.004	1.61 (.59–5.01)	.36
HIV infection	No	Ref	Ref	Ref	Ref
	Yes	6.32 (2.36–16.66)	<.001	5.68 (2.12–15.30)	<.001
Hypertension	No	Ref	Ref	Ref	Ref
	Yes	4.25 (1.61–9.39)	.001	.58 (.14–3.66)	.52
DM	No	Ref	Ref	Ref	Ref
	Yes	9.75 (2.85–25.33)	<.001	8.21 (1.38–34.00)	.02
Disease severity at admission	No dehydration	Ref	Ref	Ref	Ref
	Some dehydration	9.48 (2.80–59.16)	.002	4.06 (.90–38.28)	.07
	Severe dehydration	16.01 (4.68–100.22)	<.001	5.97 (1.29–56.94)	.02

Abbreviations: CI, confidence interval; DM, diabetes mellitus; HIV, human immunodeficiency virus; OR, odds ratio; PLR, penalized likelihood regression; Ref, reference.

^a^ORs were calculated for binary analysis using a generalized linear model and PLR. Differences were considered statistically significant at *P* < .05. The length of hospitalization was not available for 1058 patients, and clinical outcome (ie, death or discharge) was not available for 638 (Supplementary Table 1).

### Human Resources in the CTCs

The temporal distribution of healthcare workers in the community and referral CTCs during the outbreak is summarized in Supplementary Figure 1. The numbers of physicians and nurses increased significantly in the Heroes CTC, but they did not change significantly in other CTCs throughout the outbreak.

## DISCUSSION

We conducted this descriptive study for the clinical features and outcomes of patients admitted with cholera in 8 treatment centers in Lusaka, documenting the largest cohort of patients admitted with cholera. As most documented literature during cholera outbreaks focuses on epidemiological patterns and not patient-specific outcomes, this study sought to build on the growing evidence examining patient's clinical characteristics. While the overall case fatality at 1.4% was higher than the recommended <1% from the Global Task Force on Cholera Control [2], we were able to highlight the risk of adverse patient outcomes particularly in those with comorbid conditions, such as HIV and DM.

The mean age for decedents was 38 years, compared with 30 years for survivors , corresponding to previous demonstrations of older age associated with higher odds of death from cholera [2, 14]. Similarly, male patients had higher proportions of admissions and deaths than female patients. While death from cholera and sex have been shown previously to have mixed associations, it is postulated that men have a propensity to seek care later [2, 14]. Delayed care seeking by men during outbreaks has been shown to be a mortality risk factor [15].

Our findings align with previous research showing that patients with more severe dehydration are often associated with CTCs and hence with higher mortality rates [16, 17]. The highest mortality rate in our setting was seen at the LMUTH (4.3%). However, it must be noted that that this was the treatment center designated for patients with comorbid conditions and more complicated care, and hence it was better equipped to diagnose these conditions. Thus, with the selection bias in this CTC’s receipt of more complicated cases, it is not surprising that its CFR was higher. During this outbreak the highest proportion of cholera-related deaths was in the community [18, 19].

Multimorbidity is increasingly being recognized for adverse patient outcomes in acute care settings in Africa, particularly in outbreak settings such as the coronavirus disease 19 (COVID-19) pandemic, where we saw an increase in deaths due to DM and hypertension in Zambia [20, 21]. One previous publication from Iraq documented outcomes of DM and hypertension among patients with cholera in low-income settings—particularly in a conflict zone [17]. Likewise, they found a higher male predominance among patients and longer hospitalizations. Incident DM after a cholera attack has been documented as an increasing phenomenon [22].

Data from the current study also show that cholera treatment algorithms should include opt-out testing for HIV, especially in high-incidence settings such as Zambia. Patients at LMUTH had 32% HIV prevalence, compared with a general HIV prevalence of 11.1% in Zambia ( [23]. HIV status was not tested at the community treatment centers and may be a contributing factor for the escalation of care from plan A or B to plan C with transfer to the referral centers.

We demonstrate also how the deployment of additional healthcare workers during the peak of the outbreak ensured improved clinical care and survival in admitted patients. While Zambia's physician-to-patient ratio stands at 1:12 000, far below the ideal 1:5000, and the nurse-to-patient ratio is 1:14 960, compared with the recommended 1:700 [24], the outbreak saw the rapid recruitment of volunteer healthcare workers (ie, registered healthcare workers, such as physicians and nurses, without employment by the governmental health facilities at the time of the outbreak). Rosters and staff rotation with unemployed volunteers, which was listed and organized by the case management pillar of the national cholera IMS by the early phase of the outbreak, were essential for ensuring staffing to meet the increasing number of patients and have been seen during outbreaks elsewhere [25]. Recruiting 4000 additional health personnel marks a positive step toward strengthening resilience, but staffing levels remain inadequate. These disparities highlight the critical need for sustained investment in human resources to address existing gaps and enhance preparedness for and case management during future public health emergencies.

The limitations of the current study are largely attributable to its retrospective design, which has a greater propensity for missing data. Outcomes were not available for >30% of cases, and a limited number of fatal cases resulted in a wide CI in the multivariate analysis. The number of fatal cases was not significantly different from the aggregated numbers reported from the facilities during the outbreak; therefore, cases without information as to outcome were assumed to be mostly discharged cases (data not shown). This lack of information was attributed to the overwhelmingly large number of patients against the limited number of healthcare workers in the CTCs during the outbreak. In addition, as the number of vaccinated patients was limited and the dates of vaccination were not collected in the study, we could not draw conclusions regarding the impact of oral cholera vaccines on clinical outcome. Furthermore, common complications, such as fluid overload, electrolyte imbalances, and arrythmias were investigated only in the patients in critical condition or with decreased urine output and not routinely due to resource limitations. It is similarly not clear how many patients required organ support. HIV testing was conducted on an opt-in and not an opt-out basis per national guidelines, accounting for the higher proportion of HIV-positive patients identified at the tertiary centers compared with the community treatment centers.

In conclusion, in adult patients managed for cholera at community and referral treatment centers, HIV status and DM are associated with higher mortality rates. Investigation for HIV infection, comorbid conditions, and complications such as electrolyte imbalances should be instituted in testing algorithms in an opt-out fashion—even in emergency/outbreak settings. Adequate resources for supportive investigations should be included in preparedness plans, beyond simple fluid resuscitation for patients with cholera.

## Supplementary Material

ofaf277_Supplementary_Data

## Supplementary Data

Supplementary materials are available at *Open Forum Infectious Diseases* online. Consisting of data provided by the authors to benefit the reader, the posted materials are not copyedited and are the sole responsibility of the authors, so questions or comments should be addressed to the corresponding author.
