# Supplementary material for: Clinical Characteristics of 6578 Adult Patients With Cholera Admitted to Community and Referral Cholera Treatment Centers in Lusaka, Zambia, October 2023 to April 2024
Source: Open Forum Infect Dis. 2025 May 8;12(6):ofaf277. doi: 10.1093/ofid/ofaf277 (PMC12130790; doi:10.1093/ofid/ofaf277)
Supplement: ofaf277_Supplementary_Data [file ofaf277_supplementary_data.zip › NBEWEN_Supplementary_R1.docx]

**Supplementary tables**

Supplementary table 1. Number of patients hospitalized at the referral cholera treatment centers with available clinical data

| Characteristic | | | Total adult patients  (n=6,578)  [number (%)] | Community CTCs  (n=2,913)  [number (%)] | Referral CTCs  (n=3,665)  [number (%)] |
| --- | --- | --- | --- | --- | --- |
| Age | | | 6,578 (100) | 2,913 (100) | 3,665 (100) |
| Sex | | | 6,564 (99.8) | 2,905 (99.7) | 3,659 (99.8) |
| Underlying medical conditions | Human immunodeficiency virus positives | | 3,064 (46.6) | 1,651 (56.7) | 1,413 (38.6) |
|  | Hypertension | | 6,578 (100) | 2,913 (100) | 3,665 (100) |
|  | Diabetes mellitus | | 6,578 (100) | 2,913 (100) | 3,665 (100) |
| Oral cholera vaccine prior to admission | | | 3,300 (50.2) | 1,790 (61.4) | 1,510 (41.2) |
| History of present illness | | | 4,830 (73.4) | 2,602 (89.3) | 2,228 (60.8) |
| Symptoms at admission | | | 6,578 (100) | 2,913 (100) | 3,665 (100) |
| Vitals at admission | | Temperature | 3,920 (59.6) | 2,136 (73.3) | 1,784 (48.7) |
|  |  | Pulse rate | 3,964 (60.3) | 2,080 (71.4) | 1,884 (51.4) |
|  |  | Respiratory rate | 3,641 (55.4) | 1,874 (64.3) | 1,767 (48.2) |
|  |  | Systolic blood pressure | 3,268 (49.7) | 1,857 (63.7) | 1,411 (38.5) |
|  |  | Diastolic blood pressure | 3,256 (49.5) | 1,853 (63.6) | 1,403 (38.3) |
|  |  | SpO_2_ | 2,118 (32.2) | 1,229 (42.2) | 889 (24.3) |
| Disease severity at admission | | | 5,917 (90.0) | 2,863 (98.3) | 3,054 (83.3) |
| Initial treatment plan at admission | | | 5,882 (89.4) | 2,889 (99.2) | 2,993 (81.7) |
| Outcomes | | | 5,020 (76.3) | 2,446 (84.0) | 2,574 (70.2) |

Number (%) of pediatric patients with the characteristics are shown.

Supplementary table 2. Assessed clinical severity and the initial treatment plan at admission among adult patients hospitalized at the community and referral cholera treatment centers in Lusaka, between October 15, 2023 and April 30, 2024

| CTC category | Treatment plan at admission | Clinical severity at admission | | | |
| --- | --- | --- | --- | --- | --- |
|  |  | No dehydration | Some dehydration | Severe dehydration | Unknown |
| Community | A | 967 (97.1) | 11 (1.0) | 4 (0.5) | 7 (14.0) |
|  | B | 23 (2.3) | 1,057 (97.3) | 10 (1.3) | 14 (28.0) |
|  | C | 4 (0.4) | 17 (1.6) | 766 (98.1) | 9 (18.0) |
|  | Unknown | 2 (0.2) | 1 (0.1) | 1 (0.1) | 20 (40.0) |
|  | Total | 996 (100) | 1,086 (100) | 781 (100) | 50 (100) |
| Referral | A | 805 (81.1) | 92 (5.9) | 9 (1.8) | 36 (5.9) |
|  | B | 68 (6.9) | 1,387 (88.9) | 29 (5.8) | 64 (10.5) |
|  | C | 7 (0.7) | 33 (2.1) | 457 (91.0) | 6 (1.0) |
|  | Unknown | 112 (11.3) | 48 (3.1) | 7 (1.4) | 505 (82.7) |
|  | Total | 992 (100) | 1,560 (100) | 502 (100) | 611 (100) |

Number (%) of pediatric patients with the selected treatment plan at admission in each of the clinical severity at admission are shown.

Supplementary table 3. Case fatality rate among adult patients hospitalized at the cholera treatment centers in Lusaka, between December 1, 2023 and March 31, 2024

| Characteristic | Case fatality rate (%) | Number of fatal cases | Number of patients |
| --- | --- | --- | --- |
| Total | 1.0 | 51 | 5,020 |
| CTC category |  |  |  |
| Community | 0.7 | 17 | 2,446 |
| Referral | 1.3 | 34 | 2,574 |
| Facility |  |  |  |
| Community CTC (A) | 0.4 | 1 | 258 |
| Community CTC (B) | 0 | 0 | 495 |
| Community CTC (C) | 1.8 | 9 | 491 |
| Community CTC (D) | 0 | 0 | 90 |
| Community CTC (E) | 1.2 | 7 | 516 |
| Community CTC (F) | 0 | 0 | 594 |
| Referral CTC (Heroes Stadium) | 0.1 | 2 | 1,802 |
| Referral CTC (LMUTH) | 4.1 | 32 | 772 |
| Sex |  |  |  |
| Female | 0.5 | 11 | 2,115 |
| Male | 1.4 | 40 | 2,896 |
| Underlying conditions |  |  |  |
| Human immunodeficiency virus positives | 2.5 | 8 | 324 |
| Hypertension | 3.8 | 6 | 157 |
| Diabetes mellitus | 8.5 | 4 | 47 |
| Epilepsy / seizures | 0 | 0 | 8 |
| Tuberculosis | 4.3 | 1 | 23 |
| Liver diseases | 25.0 | 2 | 8 |
| Kidney diseases | 16.7 | 1 | 6 |
| Alcohol dependency | 25.0 | 1 | 4 |
| Anemia | 0 | 0 | 3 |
| Oral cholera vaccine prior to admission |  |  |  |
| Yes | 0.5 | 1 | 222 |
| No | 0.6 | 15 | 2,504 |
| Initial diagnosis at admission |  |  |  |
| No dehydration | 0.2 | 4 | 1,607 |
| Some dehydration | 1.1 | 22 | 1,995 |
| Severe dehydration | 1.9 | 20 | 1,031 |
| Initial treatment plan at admission |  |  |  |
| A | 0.1 | 2 | 1,563 |
| B | 1.2 | 23 | 1,916 |
| C | 2.0 | 21 | 1,045 |

Case fatality rate was calculated as the number of fatal cases divided by the number of patients in the relevant group.

**Supplementary figure legends**

Supplementary figure 1. Temporal distribution of the health care workers human resources assigned to the community and referral Cholera Treatment Centers in Lusaka, Zambia, between October 2023 and April 2024
